# Microstructural Characteristics of Graded Ni-Fe Coatings Fabricated Through DED-L

**DOI:** 10.3390/ma19020271

**Published:** 2026-01-09

**Authors:** Marco Brand, Ion-Dragoş Uțu, Nicușor-Alin Sîrbu, Ion-Aurel Perianu, Denis Andrei Predu, Gabriela Mărginean

**Affiliations:** 1 Department of Materials and Manufacturing Engineering, Faculty of Mechanical Engineering, Politehnica University Timişoara, Bv. Mihai Viteazu 1, 300222 Timisoara, Romania; dragos.utu@upt.ro; 2 Institute of Mechanical Engineering, Westphalian University of Applied Sciences, Neidenburger Str. 43, 45897 Gelsenkirchen, Germany; 3 National Research & Development Institute for Welding and Material Testing (ISIM Timişoara), Bv. Mihai Viteazu 30, 300222 Timisoara, Romania; asirbu@isim.ro (N.-A.S.); aperianu@isim.ro (I.-A.P.); dpredu@isim.ro (D.A.P.)

**Keywords:** parameter study, directed energy deposition-laser, functionally graded materials, deposition strategy, powder processing, surface protection

## Abstract

**Highlights:**

**What are the main findings?**

- Bidirectional samples show fewer pores than samples with monodirectional movement.
- Increasing the scanning speed leads to lower porosity (bidirectional scanning mode with 90° layer rotation).
- Bidirectional deposition with 90° layer rotation exhibits best quality

**What are the implications of the main findings?**

- Systematic study of scanning speed and deposition strategy.
- Practical guidance for process improvement of graded metal coatings; properties under evaluation.
- Functionally graded Ni-Fe coatings fabricated by DED-L with tailored microstructures.

**Abstract:**

Directed Energy Deposition-Laser (DED-L) enables high-performance coatings through melting and successive powder deposition. Its compositional flexibility suits functionally graded layers that enhance corrosion and wear resistance. This study aimed to improve parameters for producing dense, defect-free, graded Ni- and Fe-based coatings by varying the scanning speed and deposition strategy (monodirectional versus bidirectional, with/without layer rotation), while keeping the power and hatch distance constant. Laser and electron microscopy were used to link parameters to porosity and uniformity. Optimal settings minimized pores, improved interlayer bonding and preserved geometry; inadequate parameters yielded porous, irregular deposits. A bidirectional path with 90° rotation appeared best. Ongoing research activities are needed to assess its properties.

## 1. Introduction

Components operating at elevated temperatures as in the chemical processing industries, aerospace or power generation, necessitate surfaces that exhibit resistance to oxidation, corrosion and wear. These surfaces must also maintain structural integrity and facilitate cost-effective repair. Nickel-based superalloys continue to be regarded as the standard for such environments; nevertheless, their performance is increasingly evaluated in relation to supply risks and sustainability objectives. Nickel has historically been utilized primarily in stainless steel applications [1,2]; however, a considerable increase in demand has been witnessed in the battery sector [3,4]. The application of Nickel superalloys [5,6,7], while comparatively less extensive, remains of paramount strategic significance.

In contrast to the study on critical raw materials (CRMs) for the European Union published in 2020, the European Commission included nickel in their study from 2023 [8]. Even though nickel has not yet fallen below the threshold value that would necessitate its classification as a CRM, it is nevertheless considered as a strategic raw material (SRM). The sustainable and economical use of SRMs and CRMs have gained significance and will continue to be of central importance in the future due to their significance in essential technologies and vulnerability to supply risks [9]. On 8 March 2022, the London Metal Exchange suspended nickel trading for the first time in history in response to serve price fluctuations triggered by a record 250% increase within a single day, thereby exemplifying the metal’s volatility [10]. Consequently, it is imperative that nickel is conserved in current production, to prevent a future scarcity of resources. A key focus of current research is to replace CRMs and SRMs, respectively, or to reduce their usage.

Additive manufacturing technologies have become increasingly prevalent in the production of high value components, and are also attracting considerable interest in the coating industry due to their resource-efficiency and versatility [11,12,13]. In the Directed Energy Deposition-Laser (DED-L; also known as Laser Metal Deposition (LMD), Laser Cladding (LC), Laser Energy Net Shaping (LENS^TM^) [14]) process, the feedstock (powder- or wire-shaped) is fed to a laser-generated melt pool, where it is deposited according to a predefined tool path. The process enables metallurgical bonding, multi-axis build, high deposition rates, and localized refurbishment beyond the reach of conventional coating technologies. Wire-based DED-L is suitable for medium to large-scale components of simple complexity. Since they do not pollute the environment, they also enable the coating when the use of powder-based materials is limited due to lack of safety equipment [14,15,16]. Powder-based DED-L is used for complex components with high-precision requirements. The possibility of using a multi-nozzle or multi feeding system makes powder-based DED-L suitable for functionally graded materials (FGMs) [13,14,17]. Whereas in a wire-based system, a seamless transition between the individual materials is challenging [18].

FGMs outline multi-material layer structures which are applied in predefined proportions. This approach aims to combine the advantages of the materials involved, enabling tailored properties to specific-use cases beyond the limitations of ‘traditional’ materials [18]. A chronological overview of research on FGMs can be found in the work of Alkunte et al. [18] or Nazir et al. [19]. Research on FGM ranges from the first experiments in the 1980s, through numerous patents, conferences and scientific papers, to their central role in today’s high-performance applications [18,19]. In addition to combining the advantages of different materials, FGMs also offer the possibility of saving raw materials, whereas CRMs are part of current FGM research activities such as cobalt FGMs [20,21,22]; titanium FGMs [23,24,25]; aluminum FGMs [26,27,28]; or copper FGMs [29,30,31].

FGMs comprising nickel-based superalloys and austenitic stainless steel have been researched owing to their combined advantages of excellent strength, ductility, and corrosion resistance [32,33,34,35]. Nickel-based superalloys attain their strength through precipitation hardening, whereas the austenitic stainless steel provides good ductility due to its single FCC structure [32]. This balanced combination enables application from cryogenic up to high temperatures, ideal for nuclear reactors and aerospace or automotive purposes [32,33,34]. In their work, Sagong et al. [32] produced a rectangular block of 8 mm thickness. First, they deposited 27 layers of SS316L, followed by 27 layers of Inconel 718. They used a travel speed of 800 mm/min, a bidirectional scanning pattern, and a 90° layer rotation for the scanning strategy. Within the 500 µm thick gradient material zone at the 316L/Inconel 718 interface, fine cracks containing brittle laves and NbC were observed. These cracks were particularly prevalent near the 316L side. However, despite these cracks, the multi-material samples exhibited superior yield and ultimate tensile strength compared to values predicted by the rule of mixture. Chen et al. [33] used a different approach to fabricate the FGM components, through continuous composition variation from 100% SS316L close to the substrate to 100% Inconel 625 at the outermost layer. The FGMs exhibited a dense microstructure with continuous compositional changes and strong metallurgical bonding. Among other things, they found that increasing Inconel 625 content and heat input led to thicker primary dendrites and wider spacing, with secondary phases appearing when Inconel 625 exceeded 80%. The microhardness increased gradually along the gradient, reaching the maximum at the 50/50 mixture.

A general overview of the influence of scanning strategies and the corresponding parameters on laser-based additive structures was published by Dar et al. [17]. In the context of a graded structure comprising NiCrBSi and 316L, Banait et al. [34] varied the laser power, travel speed, and powder feed rate. The determination of the optimal parameters was based on the microstructure and track geometry. They determined that a laser energy input per length unit of >60 kJ/m results in a continuous, uniform deposition. Furthermore, they stated that the aspect ratio (track width/track height) must exceed five to eliminate inter-run porosity. Ghanavati et al. [36] in turn focused on the analysis of distortion and residual stress in SS316L-IN718 multi-material structures manufactured by DED-L. Thermomechanical finite element modeling was utilized to track defect development. Key process parameters were optimized through response surface methodology, and thermodynamic calculations were employed to explore the influence of compositional gradient designs on abrupt stress variations. Statistic modeling indicates that maximum longitudinal strain coincides with minimum residual stress under conditions of high laser power combined with low powder feed rates. Yang et al. [37] investigated defect formation in DED-L-deposited FGMs comprising SS316L and IN718, focusing on the effects of laser power and powder feed rate. They found that high laser power combined with high powder feed leads to over-deposition that exceeds the intended build height due to excessive melting. Conversely, low values of both parameters result in under-deposition. Insufficient melting was observed when low laser power was paired with high powder feed, resulting in smaller melt pools, incomplete fusion and interlayer voids. High laser power in combination with low powder feed causes gas entrapment within the melt pool, thereby leading to the formation of keyhole pores. These defect mechanisms were subsequently confirmed through experimental analysis, highlighting the pivotal role of process parameter optimization in ensuring build quality.

The increasing utilization and extensive range of applications of SRM, such as Inconel 718, renders them susceptible to being categorized as CRMs. Functionally graded material structures offer a promising solution for conserving these valuable resources by optimizing material distribution and reducing reliance on expensive or scarce alloys. Nevertheless, the development and application of stainless steel 316L-Inconel 718 graded structures remain challenging due to the lack of standardized processing protocols and the inherent complexity in characterizing multi-material interfaces. The central research question that this study seeks to address is how variation in scanning strategies—including scanning speed, deposition strategy, and interlayer deposition angle—affect the microstructure and performance of these graded coatings. The controlled manipulation of these parameters can tailor the compositional gradient and mechanical properties, thereby enhancing coating functionality while minimizing Inconel 718 usage. This work provides novel insights into stainless steel 316L-Inconel 718 functionally graded structures by systematically investigating process–structure–property relations under varied deposition conditions.

## 2. Materials and Methods

This paper investigates a graded layer structure using two commonly used powders: a Ni-based Inconel 718 powder *Osprey^®^ Alloy 718−150 +53 µm* (Sandvik Machining Solutions AB, Sandviken, Sweden; abbreviated as In718) exhibiting a particle size range of 53–150 µm and a Fe-based stainless steel powder *UTP PLASweld*^TM^ *STAINLESS 18* (voestalpine Böhler Welding Germany GmbH, Hamm, Germany; abbreviated as *316L*) exhibiting a particle size range of 50–150 µm. Carbon steel plates (8 mm) were used as substrate material. Table 1 shows the chemical composition of the powders (value sourced from datasheet provided by the manufacturers) and the substrate (measured using UV spectral analysis system *SPECTROMAXx* from Spectro Analytical Instruments GmbH, Kleve, Germany).

Since the powder quality influences the quality of the deposited layer, the powders were examined from the top and cross-sectional viewpoints using a *Zeiss Gemini Sigma 300 VP* scanning electron microscope (SEM; Carl Zeiss AG, Oberkochen, Germany).

**Figure 1:**
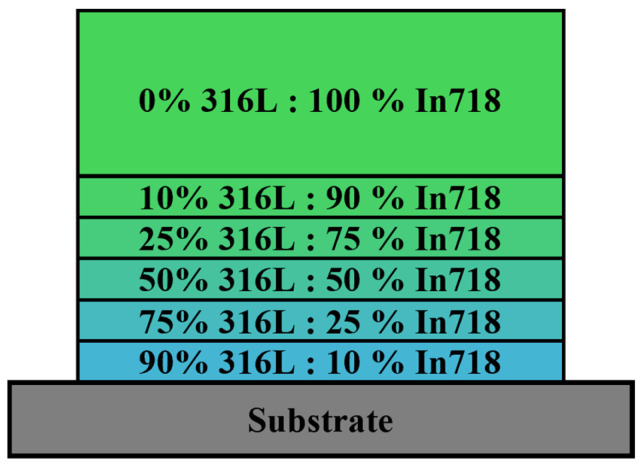
Graded layer structure of Directed Energy Deposition-Laser (DED-L) samples, with color gradients representing compositional variations in 316L to In718 ratios.

The graded layer structure manufactured in this study is shown in Figure 1. The high 316L content in the bottom layer was selected to achieve a good adhesion to the substrate (similar E-modulus, no additional brittle phases), while the gradual increase in In718 content in the subsequent layers creates a smooth transition to the In718 top layers. Four layers were deposited for the 100% In718 top layer (all other ratios were deposited with a single layer).

For the deposition the DED-L system *InssTek DMT 3D Metal Printer—MX mini with PCM-Multi powder feeder* (InssTek, Inc., Daejon, Republic of Korea), was used. The feeder determines the powder throughput of the respective containers in real time using high-precision scales and adjusts the feed rate and ratio as needed. An initial feed rate test series was carried out to determine the feedability of the powders. Different powder feed rates were used to deposit a single line (the other parameters were kept constant). Since the powder feed rate is determined gravimetrically the critical ratio between the two powders is the 10% to 90% ratio. During powder profiling the stainless steel powder showed higher sensitivity to feed rate changes, therefore the 10% 316L to 90% In718 ratio was used to determine the appropriate feed rate. The difference between the set and the actual values were utilized to determine the range of possible feed rates. The results of the feed rate test are shown in Table 2.

A ratio of 10% 316L to 90% In718 for the powders used requires a feed rate >0.5 g/min and <3.0 g/min. For environmental reasons, the lowest suitable feed rate of 1.0 g/min was selected. Repeating the measurement with a 90%316L to 10% In718 confirmed the selection of 1.0 g/min.

The investigation of the graded layers is divided into two sections, as follows:

1. Preliminary parameter study to determine suitable deposition parameters and improvement of those parameters varying the scanning speed and deposition strategy (scope of this work);
2. A detailed analysis of the graded layer structure regarding their wear and corrosion behavior (subject of ongoing experiments, not part of this work).

In the preliminary experiments the graded layer structure was deposited on a 15 × 20 mm area of carbon steel substrate. The scanning speed, the angle between the individual layers, and the scanning strategy were varied individually. The deposition parameters are displayed in Table 3.

To improve the geometrical accuracy of the produced layers, the contour strategy varied between the individual layers. For the first layer, the surface contour was coated and then filled (contour–fill). For the second layer, the contour was coated first, then the infill was coated, followed by another contour coating (contour–fill–contour). The deposition direction of the contour changed with each pass. This sequence was repeated for subsequent layers.

**Figure 2:**
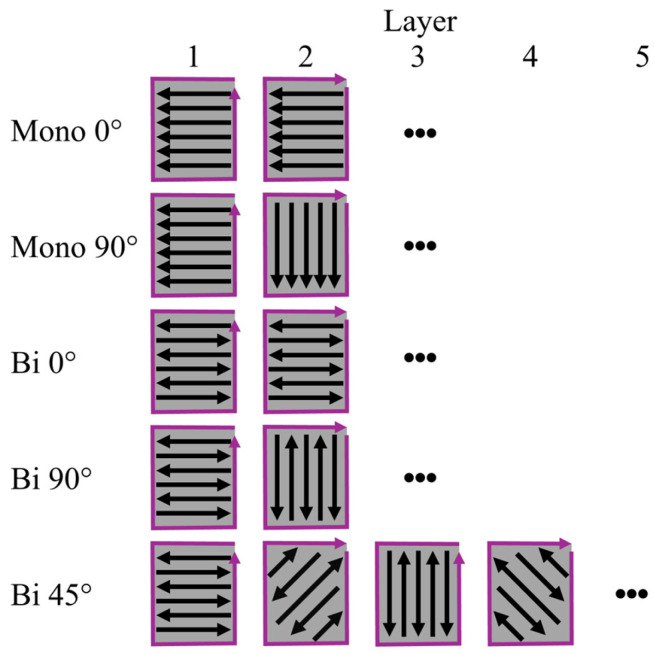
Layered deposition of different strategies with contour path (purple) and infill direction (black) for deposited functionally graded material (FGM) layers.

Figure 2 shows the different deposition strategies examined in this study. These strategies result from the deposition strategy method (monodirectional or bidirectional) and the angle between the individual layers (0°, 45°, or 90°).

Optimal coating parameters are defined by their ability to produce a dense, defect-free layer structure that exhibits strong adhesion to the substrate. Such coatings demonstrate a high degree of hardness at the outermost surface thereby enhancing wear resistance. Concurrently, they maintain a comparatively softer internal hardness, thus improving ductility and accommodating mechanical stresses. Initially, a systematic examination of the as-printed surface was conducted to determine quality surface roughness and identify excessive morphological irregularities that could require increased surface-finishing processes. A detailed metallographic analysis of the samples’ cross-sections was subsequently conducted with the objective of detecting manufacturing-induced defects including porosity, microcracks, and lack of fusion. Specimens which demonstrated a defect-free microstructure were considered for further evaluation. This further evaluation encompassed adhesion to the substrate and hardness profile across the coating thickness. The determination of optimal coating parameters was thus predicted on these rigorous criteria—emphasizing reduced surface roughness, absence of internal defects, and favorable mechanical properties—to ensure robust coating performance and structural integrity. Samples that satisfied all the stipulated requirements were considered suitable for microstructural analysis and energy dispersive X-ray spectroscopy (EDS) analysis.

To determine the best parameter combination, the layers produced were evaluated using the following methods. First, the as-deposited structure was evaluated microscopically in top-view using a confocal scanning laser microscope (CLSM) *KeyenceVK-X200* with a *Keyence VK-X250* control unit (Keyence Corporation, Osaka, Japan).

In other studies, cracks were found within the graded layers [32,36,37,38,39]. The tendency to crack formation was estimated according to Schwanekamp et al. [40] using top-view measurement. Although this methodology is primarily adapted for brittle materials such as ceramics, intermetallic phases and chromium carbide can also cause cracking. The samples were metallographically prepared (320-500-1000-2000-4000 grit SiC-paper) to obtain a uniform surface and to visualize any possible cracks. The resulting imprints (HV30, holding time 15 s) were evaluated using CLSM.

The microstructure and any process-related defects (such as pores and cracks) were evaluated in metallographically prepared (320-500-1000-2000-4000 grit SiC-paper; 3 µm diamond suspension) cross-sections using CLSM. To ascertain the present porosity, the CLSM images were evaluated using *ImageJ* (version 1.53k; developed by Wayne Rasband at the National Institutes of Health in Bethesda, MD, USA) within a rectangular area of equal size (measuring 1000 × 600 pixels, which is approximately 1350 µm × 825 µm). The images were converted into binary images, and the porosity was determined using the *Threshold* function based on the greyscale (pores appear black due to the depression, while the metallic matrix and precipitations corresponds to lighter greyscales).

The adhesion between the coating and the substrate was determined using a *KB 250* Brinell hardness tester (KB Prüftechnik GmbH, Hochdorf-Assenheim, Germany). A 2.5 mm diameter indenter was applied at the interface between the substrate and the coating at a load of 62.5 kp (maximum load after 5 s; holding time 15 s). Then the indentation and the interface were examined for cracks or delamination using the CLSM.

Microhardness distribution was performed to determine the hardness profile across the deposited layers cross-sectionally. The indenter was applied to the samples for 10 s according to the HV1 load level using *ZHVµ* (ZwickRoell GmbH & Co. KG, Ulm, Germany) microhardness tester.

To evaluate the element distribution of the best parameter combination, an EDS analysis was performed in the SEM. The samples were embedded in cross-section using thermoplastic resin and metallographically prepared (320-500-1000-2000-4000 grit SiC-paper; 3 µm diamond suspension). Finally, the microstructure was determined in the CLSM, using samples etched with V2A-etchant.

## 3. Results and Discussion

**Figure 3:**
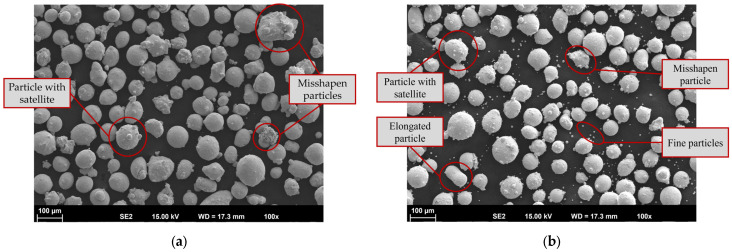
Scanning electron microscope (SEM) top-view images of (**a**) Fe-based 316L and (**b**) Ni-based Inconel 718 powder particles.

To interpret subsequent material behavior and performance, it is essential to possess a comprehensive understanding of the powder characteristics. Initial analysis of the morphology and distribution of the powder particles was performed from both top-view and cross-section perspectives. The observations presented herein yield valuable insights pertaining to dimensions, morphology, density, and layering uniformity of the particles under scrutiny. Figure 3 shows the top-view SEM images of both powders.

**Figure 4:**
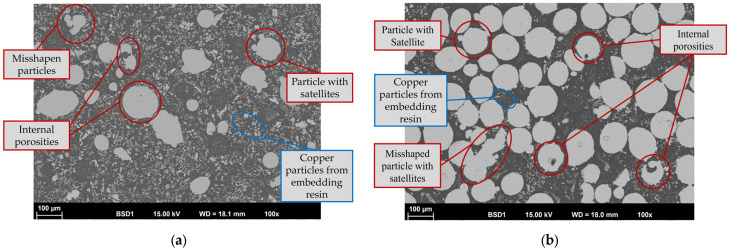
SEM cross-section images of (**a**) Fe-based 316L and (**b**) Ni-based Inconel 718 powder particles (copper particles from embedding resin to enable conductivity for SEM analysis).

Most of the particles exhibit a spherical or nearly spherical shape. A spherical particle shape is beneficial for uniform deposition due to, e.g., better inflight behavior and uniform melting. Nevertheless, both powders also include irregularly shaped particles, spherical particles with satellites (fine particles that adhere to larger particles) and fine particles. The latter are more common in the Inconel 718 powder. These features, along with other factors, negatively impact flowability and can lead to defects caused by uneven melting. The particles compactness and internal porosity were examined using metallographically prepared cross-sections (see Figure 4).

As shown in Figure 4a, the satellites are metallurgically bonded to the corresponding large particles. Only a very small fraction of the 316L particles showed internal porosity. In comparison Inconel 718 powder has significantly more internal porosity. However, these particles with internal porosity are quite uncommon; therefore, the powders are regarded as having a suitable compactness. Despite the necessity of processing both powders through sieving or sifting, and potentially recompacting, no processing was considered for the present work. This decision was based on the fact that the *as-delivered* condition of the powders aligns more closely with industrial practice, enabling a more realistic simulation of the application. The processing of powders is not infeasible in industrial applications, particularly in time-critical scenarios such as the repair work of worn components, due to the substantial time investment required for processing. It is important to note that the omission of powder conditioning may result in limitations in the reproducibility between different powder batches or suppliers, due to variations in particle morphology and distribution. It is evident that powder processing techniques, such as sieving or recompacting, have the capacity to enhance homogeneity and reduce variability. However, considering the objective of simulating standard industrial practice—wherein powder conditioning is frequently excluded due to time constrains or expenses—it is hypothesized that such processing may result in only slight enhancements in porosity, without inducing substantial alterations in the overall microstructural integrity or functional performance of the coating. Nevertheless, targeted studies incorporating systematic powder conditioning could provide a deeper understanding of its influence on process reproducibility and further influences on coating properties.

The samples (Table 4). were deposited according to the methodology described in the second chapter.

In the first step, the scanning speed of 400 mm/min was selected and the angle between the deposited layers (0°, 90°, 45°) and the deposition strategy (monodirectional, bidirectional) were varied. Since the manufacturing process of the bidirectional strategy appeared significantly more stable than the monodirectional method in the first samples, the monodirectional strategy was not considered further.

**Figure 5:**
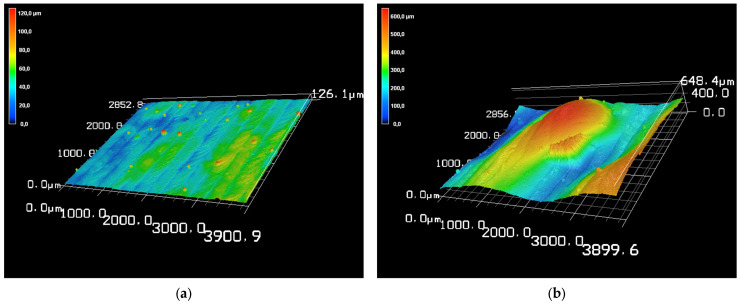
Confocal scanning laser microscope (CLSM) top-view image in 3D perspective of (**a**) FG-2 example for a suitable sample and (**b**) FG-7 unsuitable sample.

A comprehensive CLSM top-view analysis was performed to characterize the surface morphology of the samples in as-deposited condition. CLSM top-view imaging was used to quantify and evaluate the microstructural features and surface topography of the samples. Almost all samples exhibited a uniform as-deposited surface, with varying degrees of path structure visibility. Additionally, local powder particles resulting from adjacent layers overspray were present on the surface (see an exemplary structure in Figure 5a). Sample FG-7, however, exhibits a highly irregular structure with significant variations (see Figure 5b).

Although coatings are usually processed to achieve a surface finish in industrial practice, such large differences would require considerable additional effort. In addition, a significant amount would have to be removed to compensate for the irregularities, which contradicts the aim of saving resources through FGM use. Taking these factors into account, as well as the fact that the FG-7 layer thickness is almost twice that of the others (see Table 4), it was determined that FG-7 was an unsuitable parameter combination for the intended FGM deposition.

Following the surface morphology analysis via top-view CLSM images, an investigation into the crack tendency was conducted. Within the measurements no cracks were observed at the imprints or in other areas. Accordingly, none of the produced layers show a tendency to crack according to the methodology described by Schwanekamp et al. [40]. However, it should be noted that this method is intended for use with ceramic–metallic coatings. Based on these results, it can therefore only be assumed that the internal stresses do not promote cracking of the coatings. Further investigations are necessary to determine the exact level of residual stresses (not part of the scope of this work).

While top-view CLSM images and crack-tendency measurements provide valuable insight into the surface morphology and crack tendency of the deposited layers, cross-sectional examination is required to gain a thorough understanding of their internal structure and thickness. Subsequently, SEM cross-section analysis was conducted to evaluate layer uniformity, interface quality and presence of any defects (such as cracks or porosity).

**Figure 6:**
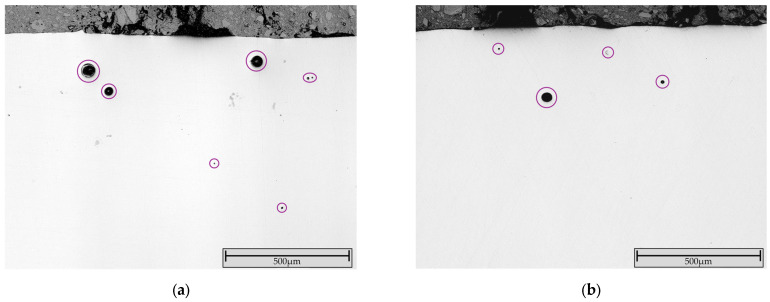

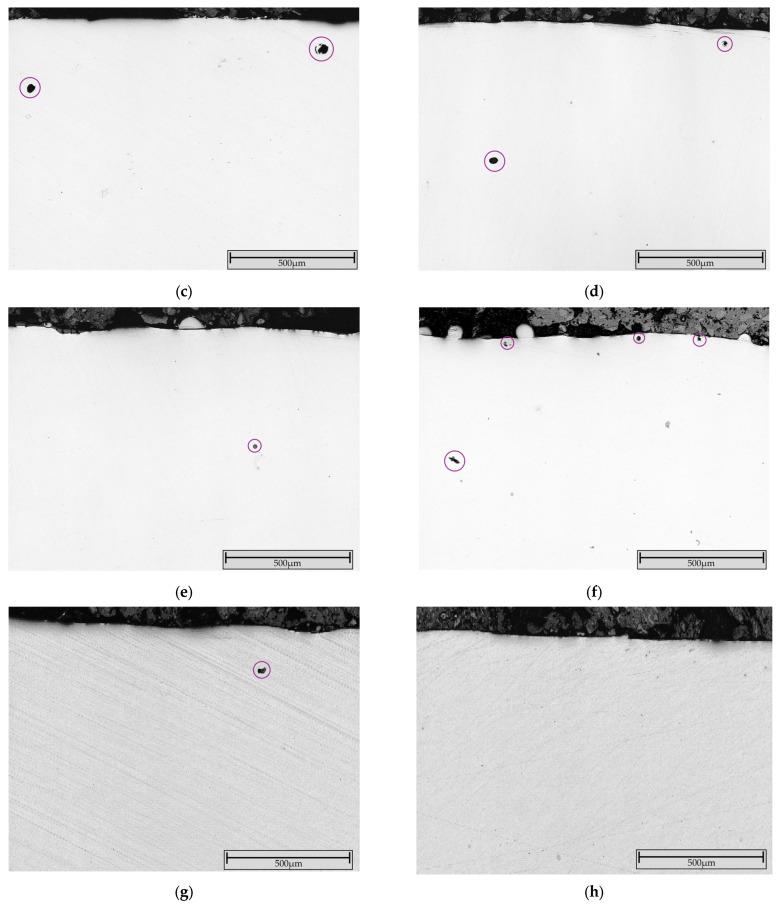

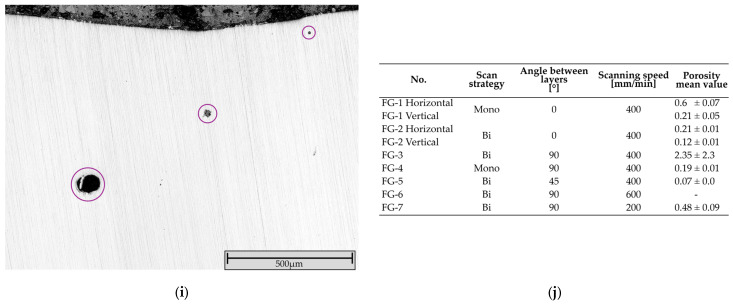
Unetched CLSM cross-section image of (**a**) FG-1 horizontal (within one track; monodirectional, 0°, 400 mm/min); (**b**) FG-1 vertical (several tracks; monodirectional, 0°, 400 mm/min); (**c**) FG-2 horizontal (within one track; bidirectional, 0°, 400 mm/min); (**d**) FG-2 vertical (several tracks; bidirectional, 0°, 400 mm/min); (**e**) FG-3 (bidirectional, 90°, 400 mm/min); (**f**) FG-4 (monodirectional, 90°, 400 mm/min); (**g**) FG-5 (bidirectional, 45°, 400 min/min); (**h**) FG-6 (bidirectional, 90°, 600 mm/min); and (**i**) FG-7 (bidirectional, 90°, 200 mm/min). Porosity is circled in purple and dark gray spots inside structure are precipitations. (**j**) Table with correlating manufacturing parameters and mean porosity value.

For the samples with 0° layer shifting (FG-1 and FG-2), a comprehensive microstructural evaluation was conducted to discern potential variations, encompassing both horizontal and vertical dimensions. This is not necessary for the other samples due to the rotation of the deposition. Figure 6 shows an overview of all samples investigated in unetched cross-section.

The samples exhibiting 0° layer rotation (FG-1 and FG-2) showed more porosity within a deposited track (horizontal; see Figure 6a,c) than between individual tracks (vertical, see Figure 6b,d). As demonstrated in Figure 6c,d, the bidirectional processing strategy yields a lower number of pores in comparison to the monodirectional deposition approach. This observation confirms the instabilities of the monodirectional deposition strategy observed during the manufacturing process. Using 90° layer rotation instead of 0° resulted in a reduction in porosity. This observation was derived in both bidirectional (Figure 6e) and monodirectional (Figure 6f) deposition strategy. A similar trend was observed when the scanning speed increased. While FG-6 (200 mm/min; Figure 6i) exhibited numerous pores, FG-3 (400 mm/min; Figure 6e) exhibited fewer pores and FG-6 (600 mm/min; Figure 6h) showed no porosity. Although according to the literature [17], an increased scanning speed should result in increased porosity due to the presence of trapped gasses or unmelted powder particles, the sample deposited with the fastest scan speed does not show porosity. It has been demonstrated that both low and high energy densities result in increased porosity, with the former causing elongated lack-of-fusion pores between tracks and the latter producing near-spherical keyhole pores within tracks. Keyhole pores are typically smaller than 100 µm in diameter, while lack-of-fusion pores vary widely in size depending on the processing conditions [41]. The cause of the increased porosity at slow scanning speeds might be due to the formation of keyhole porosity. Keyhole porosity is favored by slower scan speeds’ increased energy input [22,37,39]. Additionally, the present pore might result from internal porosity contained in the powder. However, further investigations are necessary to determine the cause more precisely. Defects such as pores or cracks can impair the performance characteristics of the coatings (such as corrosion or wear resistance). No cracks were found in any of the samples examined. Therefore, all samples with significant porosity were excluded. The results of the ImageJ porosity analysis (Figure 6j) and the CLSM analysis led to the following:

- FG-1, FG-2, FG-4, and FG-7 exhibited a significant number of pores in the outermost layer. Although some of these may be removed during postprocessing, it is estimated that a significant number will remain and result in impaired performance.
- In FG-3, no pores in the outermost layer were observed, yet a single large pore measuring >250 µm is evident in the middle of the layer (causes the high standard deviation in the porosity measurements).
- FG-5 only showed a single pore at the outermost layer, which most likely will be removed during postprocessing procedure.

Only FG-5 (single pore) and FG-6 (no porosity) were considered further, and the other samples were excluded from further consideration.

**Figure 7:**
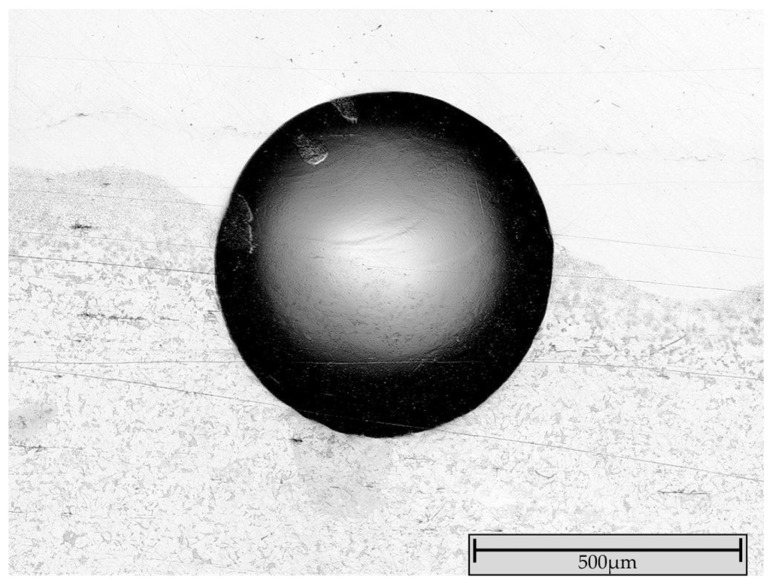
Adhesion measurement at the coating–substrate interface of FG-6 shows no delamination or cracks (the imprint on sample FG-5 also showed no cracks or delamination).

Brinell hardness measurements (62.5 kp) at the layer–substrate interface revealed no cracks or delamination (Figure 7).

This confirms the formation of a stable metallurgical bond with the substrate and supports the hypothesis based on the crack-tendency measurement, that the present internal stresses are not critical.

**Figure 8:**
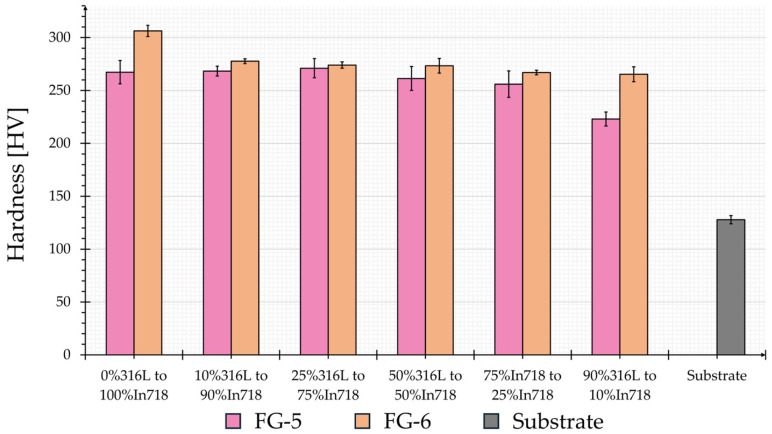
Microhardness (HV1) distribution of the individual layers (mean values) of FG-5 and FG-6 as well as of the substrate.

Microhardness (HV1) distribution was performed to evaluate the properties of the samples in more detail. The Brinell samples were etched to determine the thickness of the individual layers to ensure that measurements were taken in the center of the designated layer. Figure 8 shows the mean microhardness of the individual layers and the substrate.

Both samples exhibit significantly higher microhardness compared to the substrate. The measured microhardness values and the progression across the various 316L to Inconel 718 ratios correspond to the literature [35,37,39]. Kim et al. [39] observed cracks at a ratio of 70% 316L to 30% Inconel 718 and the 80% 316L to 20% Inconel 718. Layers. These cracks result in a microhardness drop. Although there is a slight decrease in hardness for FG-5 between 75% 316L and 25% Inconel 718 and the 90% to 10% layer, no cracks were found in the indentation or the surrounding areas. The decrease may be influenced by underlining pores, which soften the area. A general survey of all samples tested revealed that no cracks are present. The microhardness of FG-6 is higher than that of FG-5 across the entire structure and exhibits a lower standard deviation. Furthermore, the hardness distribution of FG-6 does not show any sudden drops, but rather reflects the continuous progression in hardness as indicated in the literature. Considering that FG-6 does not exhibit any pores, the sample was deemed to be the optimum of the parameter examined. Within the combinations examined in this study, a bidirectional deposition strategy with 90° shifting between layers and a scan speed of 600 mm/min achieves the best results. The laser input per unit length for this combination is 22 J/mm, which is about one-third of the 60 J/mm determined by Banait et al. [34]. However, it should be noted that they deposited a different material (NiCrBSi); additionally, they used a different powder feed rate (4 g/min versus 1 g/min in this study). With a smaller powder feed rate, a relatively large amount of energy per gram of powder is available, which ensures complete particle melting. This enables thinner layers and reduces the risk of overheating and distortion.

**Figure 9:**
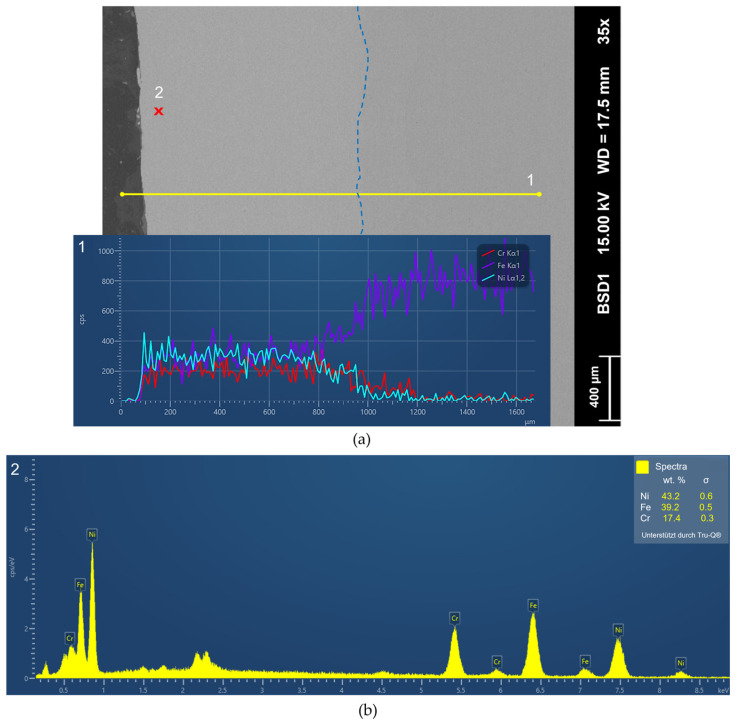
SEM analysis of optimal parameter combination of the evaluated samples (sample FG-6): (**a**) backscatter electron detector (BSD) image with energy dispersive X-ray spectroscopy (EDS) line scan (1; yellow) and corresponding diagram showing the progression of chemical composition (only Ni, Cr and Fe selected). Layer–substrate interface highlighted with blue dashed line. (**b**) EDS spot analysis of top layer (2, red cross in BSD-image (**a**)).

Following the selection of the optimal samples based on the evaluated process parameters, a compositional characterization using SEM and EDS analysis was proceeded. The line scan analysis demonstrated a uniform distribution of Ni, Cr and Fe throughout the entire thickness, indicating a homogeneous mixture (see Figure 9). An increase in Fe is evident in close to the layer–substrate interface (800 to 1000 µm), and this is attributable to the 90% 316L to 10% Inconel 718 layer and the Fe-based substrate. After the interface Ni and Cr decrease significantly; the peaks between 1000 and 1200 µm are likely attributed to dilution of the first deposited layer.

The EDS spot analysis of the top layer (Figure 9b) demonstrates a higher proportion of Fe, attributable to the mixing of the underlying layers. Although the Fe content would be reduced in thicker layers, thinner layers require less material and processing time and result in lower heat input. Furthermore, the corrosion resistance of the top layer is ensured by a Cr content that exceeds the passivation limit.

**Figure 10:**
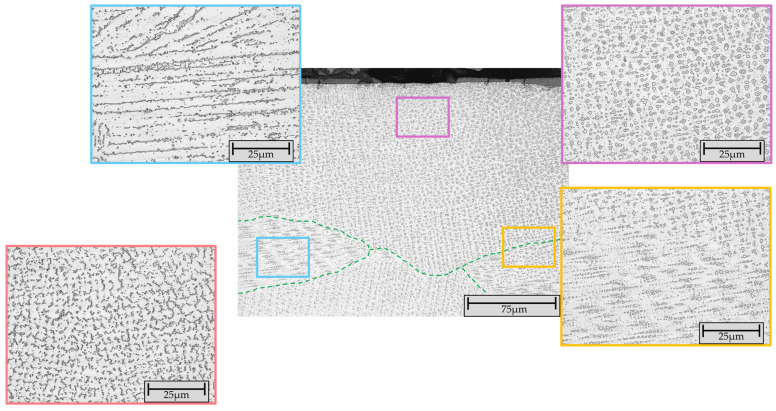
CLSM image of the etched (V2A etchant) microstructure in the top layer of sample FG-6 shows columnar dendrites (blue box), uniformly distributed globular structure (purple box) and remelted/transition zone between uniformly distributed and linear distributed globular structure (orange box). The vermicular microstructure (red box) was present in a different area it occurred in combination with a columnar dendritic structure (similar to the microstructure displayed in the blue box). The grain boundaries are highlighted with the green dashed lines.

To visualize the present microstructure in the top layer, microstructural analysis was performed on etched samples (V2A etchant) using CLSM (Figure 10).

In the top outermost layer, a uniformly distributed globular microstructure was predominantly observed. Locally, a mixture of line-shape globular structure with columnar dendrites was identified in this layer. In the underlying remelted transition areas, a linear structure of this globular microstructure or columnar dendrites were observed. A comparable microstructure has been described in the literature [35,37,39].

## 4. Conclusions

Within this work an FGM structure comprising 316L and Inconel 718 was successfully deposited using DED-L method. To ascertain the optimal parameters of the powders used, the deposition strategy, the angle between the layers, and the scan speed were varied. The analysis revealed the following:

Powder analysis revealed the presence of unsuitable particles (elongated particles, irregular particles and particles with satellites), as well as local internal porosity. However, the powder preprocessing was not considered to mimic industrial practices. Preliminary powder preparation might reveal other parameters suitability.

An initial SEM top-view analysis in the as-deposited state enabled the exclusion of one sample due to very uneven morphology (require increased postprocessing). Crack-tendency analysis and the absence of delamination and cracks during adhesion measurements indicate that the present internal stresses do not promote cracking of the coatings. Bidirectional deposition strategy, changing the angle between the adjacent layers from 0° to 90° and increasing the travel speed decreases the number of pores.

The sample with bidirectional deposition strategy, 90° layer rotation and 600 mm/min scan speed exhibited the best properties of the parameter combinations observed. Microhardness decreases slightly with increased 316L content but does not show any sudden drops or cracks at the imprints. EDS analysis of this sample showed a uniform proportion of Fe, Cr, and Ni across the layer thickness. Although a higher Fe content than specified was detected in the top layer, the Cr content is still above the passivation limit required for corrosion resistance. The outermost layer showed a predominance of uniformly distributed globular microstructure with local areas of columnar dendrites and linear globular structure.

Within ongoing research, further samples have been manufactured based on the optimal parameter combinations determined in this work. Those samples will be investigated in detail regarding the wear (e.g., sliding wear resistance and erosion wear resistance) and corrosion behavior in NaCl environments, to fully determine the potential of the FGM structures.

## Figures and Tables

**Table 1:** Chemical composition (in wt.%) of the powders and substrate (n. a. = not available, the powder manufacturers did not provide any data on the associated elements; Bal. = balance).

Component	Al	C	Cr	Fe	Mn	Mo	Nb	Ni	P	S	Si	Ti	Other
In718 powder	0.43	0.05	18.80	18.20	0.10	3.09	4.99	Bal.	0.005	0.003	0.13	1.01	n. a.
316L powder	n. a.	0.02	17.0	Bal.	0.60	2.1	n. a.	13.0	n. a.	n. a.	0.8	n. a.	n. a.
Substrate	0.06	0.18	0.043	Bal.	0.732	0.014	<0.001	0.073	0.0098	0.014	0.036	<0.0005	0.082

**Table 2:** Differences between the set feed rate and the actual feed rate value for 10% 316L to 90% In718 track deposition (with all other parameters remaining constant).

Total Feed Rate [g/min]	316L Set Feed Rate [g/min]	316L Actual Feed Rate [g/min]	316L Difference [g/min]	316L Difference [%]	In718 Set Feed Rate [g/min]	In718 Actual Feed Rate [g/min	In718 Difference [g/min]	In718 Difference [%]
0.5	0.05	0.13	0.08	160.0	0.45	0.49	0.04	8.89
1.0	0.10	0.10	0	0	0.90	0.91	0.01	1.11
1.5	0.15	0.16	0.01	6.67	1.35	1.37	0.02	1.48
2.0	0.20	0.21	0.01	5.0	1.80	1.82	0.02	1.11
2.5	0.25	0.25	0	0	2.25	2.27	0.02	0.89
3.0	0.30	0.33	0.03	10.0	2.70	2.93	0.23	8.52

**Table 3:** Deposition parameters for the graded layer structure.

Parameter	Unit	Value
Laser power	[W]	220
Powder feed rate	[g/min]	1.0
Hatch distance	[mm]	0.3
Layer height target	[mm]	0.15
Scanning speed	[mm/min]	200 400 600
Angle between layers	[°]	0 45 90
Scanning direction	-	Monodirectional Bidirectional
Shielding gas	-	Argon
Purity of shielding gas	[%]	99.9998
Gas flow rate	[L/min]	3.0
Powder feeding time ^1^	[s]	70
Between layer dwell time	[s]	60

^1^ Time to adjust powder ratios.

**Table 4:** Parameter of deposited samples and their layer thickness.

No.	Scan Strategy	Angle Between Layers [°]	Scanning Speed [mm/min]	Area Energy ^1^ [J/mm^2^]	Total Layer Thickness [µm]
FG-1	Mono	0	400	110	1428 ± 76
FG-2	Bi	0	400	110	1494 ± 35
FG-3	Bi	90	400	110	1519 ± 49
FG-4	Mono	90	400	110	1560 ± 25
FG-5	Bi	45	400	110	1547 ± 28
FG-6	Bi	90	600	73.3	990 ± 45
FG-7	Bi	90	200	220	2653 ± 70

^1^ Amount of laser energy delivered per unit of area.

## Data Availability

The original contributions presented in this study are included in the article. Further inquiries can be directed to the corresponding authors.

## Abbreviations

The following abbreviations are used in this manuscript:

BSD	Backscatter Electron Detector
CLSM	Confocal Scanning Laser Microscope
CRM	Critical Raw Material
DED-L	Directed Energy Deposition-Laser
EDS	Energy Dispersive X-ray Spectroscopy
FGM	Functionally Graded material
LC	Laser Cladding
LENS^TM^	Laser Energy Net Shaping^TM^
LMD	Laser Metal Deposition
SEM	Scanning Electron Microscope
SRM	Strategic Raw Material
